# Os odontoideum-induced sudden onset myelopathy following cervical extension injury in an adult—case report on challenges and management with 3D navigation technology

**DOI:** 10.3389/fsurg.2025.1547730

**Published:** 2025-06-18

**Authors:** Sathish Muthu, Guna Pratheep Kalanchiam, Hyun Jun Jang, Bong Ju Moon, Keun-su Kim

**Affiliations:** ^1^ Department of Spine Surgery, Orthopaedic Research Group, Coimbatore, India; ^2^ Central Research Laboratory, Meenakshi Medical College Hospital and Research Institute, Kanchipuram, India; ^3^ Department of Spine Surgery, Meenakshi Mission Hospital and Research Centre, Madurai, India; ^4^ Department of Neurosurgery, Gangnam Severance Hospital, Seoul, Republic of Korea

**Keywords:** Os odontoideum, myelopathy, vertebral artery, navigation, complex surgery

## Abstract

Os odontoideum is a relatively rare congenital anomaly of the upper cervical spine. It occurs due to developmental failure of the C2 odontoid process. Symptomatic patients develop instability resulting in cervical spinal cord compression. Surgical fixation is the management of choice in such patients to mitigate the risks of neurological worsening. On the other hand, such pathologies are challenging conditions to treat, mainly due to the surrounding delicate neurovascular structures and smaller bony anatomy of the atlas and axis. Especially in patients with variability in the normal anatomy of osseous and vascular structures, it is even more difficult to establish an effective stabilization strategy. Over the years, it has been proven that the use of pedicle screws is far superior to other techniques like sublaminar wiring in the cervical spine. However, it may not be possible in several cases due to anatomical constraints and lack of sufficient experience for early career surgeons to execute the surgical plan with ease and confidence. 3D-CT-based navigation has enabled real-time guidance for screw trajectory. They have significantly helped surgeons in the appropriate placement of surgical hardware, even with lesser surgical exposure and in minimally invasive techniques. We present the utilization of this technology in a case of sudden onset quadriparesis due to atlantoaxial instability secondary to Os odontoideum. This article highlights the effectiveness, safety, and precision of 3D-CT guidance in managing such complex case scenarios.

## Introduction

Surgical treatment of Craniovertebral junction and upper cervical instability is often demanding and rigorous owing to the vicinity of the brainstem and crucial vascular structures. Achieving a stable fixation is critical in these pathologies to ensure optimal patient outcomes. Surgeons often prefer the Goel-Harms construct as the mainstay of treatment for upper cervical instrumentation due to their proven biomechanical stability. However, it is technically challenging, especially in patients with altered and modified anatomy. Freehand placement of screws could be tricky in these patients and would result in catastrophic complications if not executed properly. Imaging-guided surgical procedures have revolutionized spine care and improved the safety of overall procedures (1).

Advancements like 3D-CT-based navigation have enabled real-time guidance for screw trajectory. They have significantly helped surgeons in the appropriate placement of surgical hardware, even with lesser surgical exposure and in minimally invasive techniques (2). The screws could be visualized in all three planes: sagittal, coronal, and axial while insertion, thereby minimizing the chances of screw breech and implant failure. This technology has aided in the confident execution of the optimal surgical plan with ease even in the hands of an early career surgeon bridging the experience gap necessary for such difficult scenarios. We would like to highlight the importance of this technology using this case report that illustrates the challenges in a complex case scenario of atlantoaxial instability with altered vertebral artery anatomy. We reported the case as per the CARE guidelines and the checklist is attached as Supplementary File 1.

## Case description

**Figure 1:**
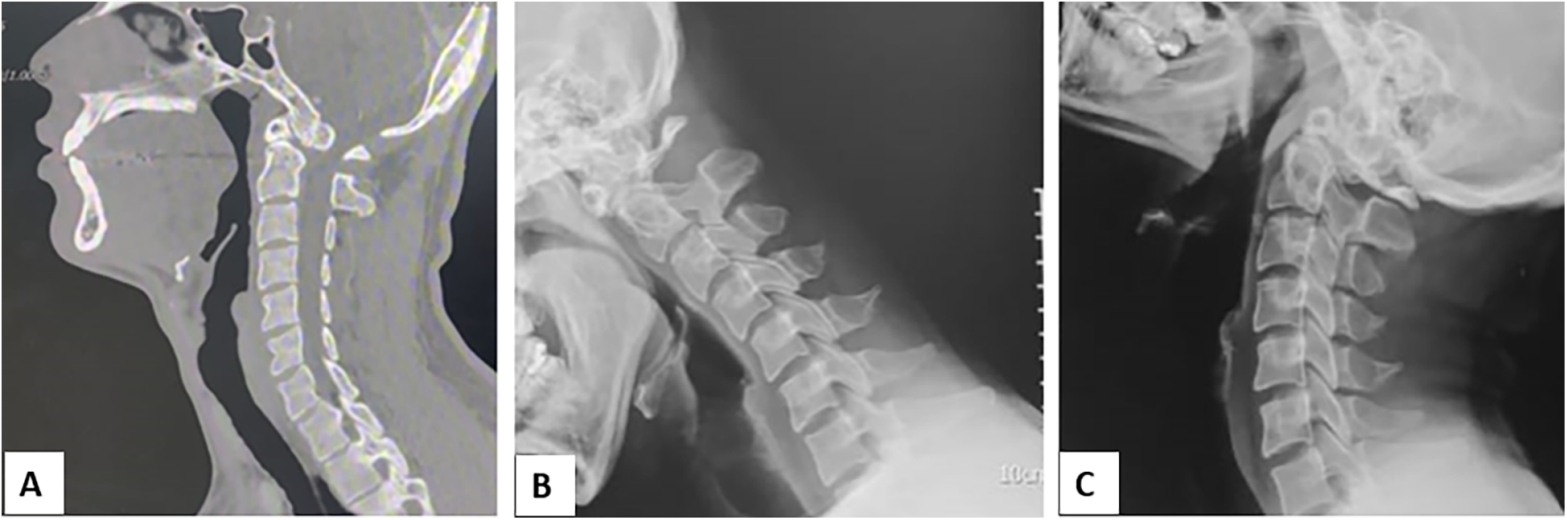
**(A)** Mid-sagittal computed tomography of cervical spine showing unfused C2 body with the odontoid process **(B,C)** and dynamic radiographs of Cervical spine showing C1–C2 instability. Three side-by-side medical images of the cervical spine. Image A is a CT scan showing detailed bone structure. Image B is an X-ray with improved contrast, highlighting vertebrae. Image C is a darker X-ray showing alignment.

**Figure 2:**
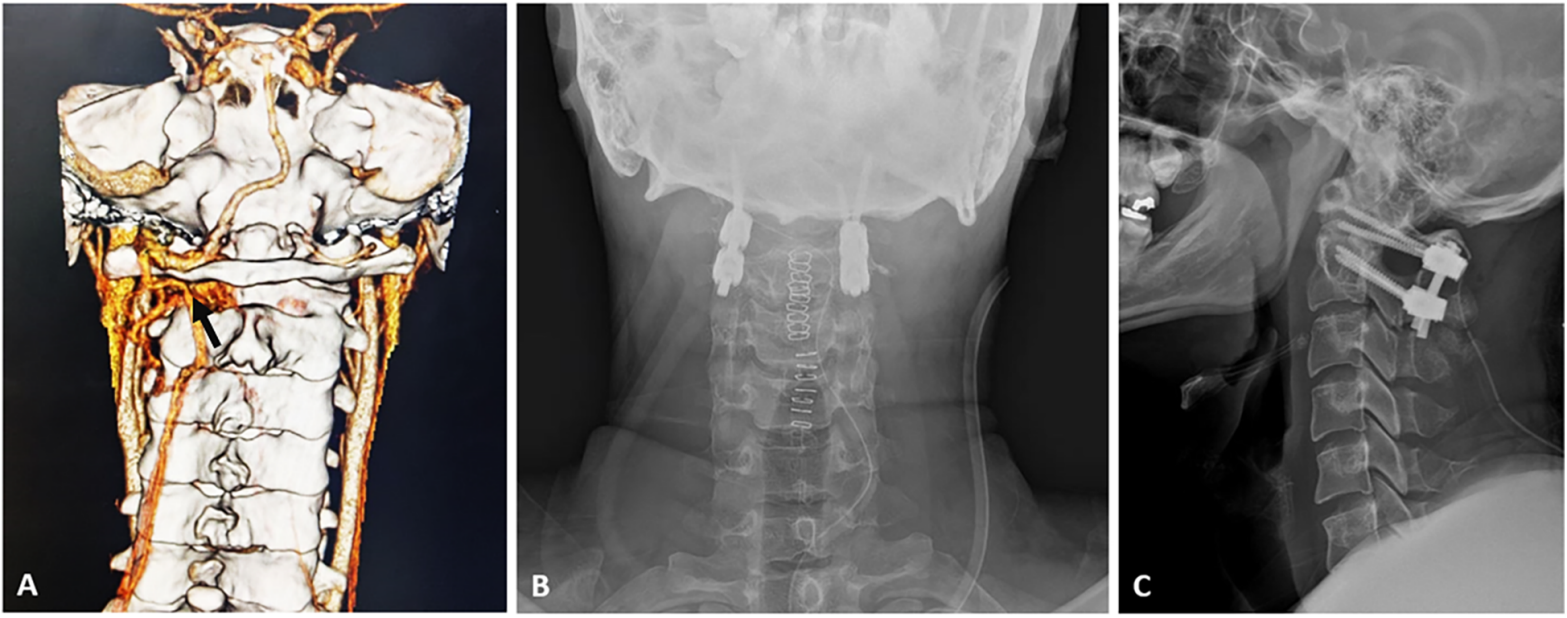
**(A)** Computed tomogram-angiography of Cervical spine showing abnormal course of left vertebral artery with tortuous and dilated vascular channels (black arrow) at C1–C2 level; post-operative antero-posterior **(B)** and lateral **(C)** radiograph showing a stable Goel-Harms construct under navigation guidance.

A 24-year-old male patient presented to the outpatient clinic with complaints of neck pain (VAS 7/10) and numbness with weakness in his hands and feet following a trivial fall and injury to the neck. There was no associated radicular pain in the limbs. On evaluation, motor power was reduced to MRC (Medical Research Council) grade: 4/5 in bilateral upper and lower limbs. The sensation was felt equally and reflexes were normal in all four limbs. Lhermitte's sign was positive and hand myelopathy signs like grip release test and finger escape sign were positive. The patient did not have any associated medical or surgical comorbid illness. Evaluation with a cervical spine radiograph and CT scan revealed an Os odontoideum at the C2 vertebra. Supervised dynamic radiographs were done and showed instability at C1–C2 (Figures 1A–C) and there was no associated Occipito-atlantal instability. Hence, surgical fixation of C1–C2 by the Goel-Harms technique was planned. However, on evaluation with CT angiography, the abnormal course of the left vertebral artery was noted with significant dilatation and tortuosity of the vessels at the C1–C2 level (Figure 2A). Hence, we decided to use 3D-CT navigation for screw placement as the dissection around the C1–C2 to visualize the anatomical landmarks would result in profuse bleeding complicating the fixation. Using the Mayfield head holder, the patient was positioned prone, and using the standard posterior midline approach, exposure was performed by subperiosteal dissection. Care was taken to avoid dissecting around the vascular channels at C1-C2. In our case, using 3D- navigation, anatomical landmarks on the navigation monitor and screws were applied. The Dynamic Reference Array (DRA) for navigation was attached to the Mayfield clamp. During surgery, no major bleeding was encountered and stable fixation at C1–C2 was achieved (Figures 2B,C). The patient was mobilized on the first postoperative day and his VAS score was reduced (1/10) and motor power improved to MRC grade 5/5 at 1 month follow-up.

## Discussion

Surgical fixation of C1 and C2 vertebrae is often challenging even for experienced surgeons, due to the proximity of vital neural and vascular structures. Various options of surgical stabilization have evolved over the years and the posterior screw fixation of C1 and C2, described Goel (3) and Harms (4), is widely accepted and performed by surgeons worldwide. This is primarily because, C1 lateral mass screw with C2 pedicle screw is considered versatile and biomechanically superior with better fixation, greater pull-out strength, and higher fusion rates (4, 5). However, variations in bony and vascular anatomy at C1–C2 make the procedure more complex. Alterations and inconsistencies in vertebral artery courses have been well-documented in the literature (6, 7). Anatomical variations concerning their origin, branches, and course though relatively uncommon, have major implications on the surgical management of upper cervical spine (8). Wakao et al. (9) observed a 10% incidence of high-riding vertebral artery in a study of 480 Japanese patients and Yeom et al. in their study reported a remarkably high risk of vertebral artery injury in such patients (10). Other less congenital variants including persistent first intersegmental artery, and perforated vertebral artery also pose a risk during C1–C2 instrumentation. In addition to the altered course, vertebral artery hypoplasia and its surgical implications have also been reported (7).

3D-CT guided navigation provides real-time guidance in multi-planar views for improved accuracy of screw placement. Another advantage of the current generation navigation systems is that CT images of different operative levels can be obtained as a single sequence, minimizing the time to acquire these images (11). Using navigated probes, the exact position and direction of the entry awl, pedicle probe, and screws could be tracked with better visualization of bony anatomical structures. In our patient, there was instability at C1–C2 owing to Os odontoideum causing upper cervical myelopathy, necessitating a stable fixation. On evaluation, the left vertebral artery was abnormal with a tortuous, dilated course, and abnormal vascular loop formation especially at the level of C1–C2. If we had planned for a free-hand C1 lateral mass screw, this would result in torrential bleeding resulting in catastrophic complications, during dissection around the C1 lateral mass. However, 3D CT navigation provided us the advantage as compared to free-hand insertion, which would have been less feasible in this case. Using navigation, we were able to identify the anatomical landmarks in C1 precisely and guide the screw placement accurately without any need for extensive dissection. The advantages of navigation guidance in C1–C2 screw placement compared to fluoroscopy guided screw placement has been enumerated in Table 1. In addition to the anticipated arterial bleeding in our case, while performing the Goel-Harm technique, the venous plexus around the C1 lateral mass tends to bleed profusely. Even with the use of hemostatic agents and mechanical compression, bleeding could obscure the vision of the operating surgeon, adding to the difficulty in instrumentation and increasing the operating time. Hitti et al. in their study of 45 patients undergoing C1–C2 fixation, compared the surgical blood loss in navigation-guided screws with non-navigated screws and observed that blood loss was significantly reduced in the navigated group without increase in the overall operative time (12).

**Table 1:** Showing the differences between 3D-Navigation guided and fluoroscopy guided posterior C1–C2 fixation techniques.

S.NO	Navigation-guided C1–C2 fixation	Fluoroscopy guided C1–C2 fixation
1	More accurate placement of screws	Relatively less accurate screw placement
2	Safety profile is high as the bony anatomy can be visualized in axial, coronal and sagittal planes during screw placement	Relatively less safe as the bony anatomy can only be visualized in anteroposterior and lateral views during screw placement
3	Can enable screw placement even in narrow osseous corridor as in anomalous anatomy	Screw placement is difficult in patients with abnormal bony anatomy
4	In patients with vascular alterations CT guided screw placement is relatively safe (as in our case)	The risk of vascular injury is high is patients with aberrant vascular structures
5	Navigation guidance enables C1–C2 surgical fixation even in less experienced surgeons with relative ease	Fluoroscopy guided screw placement is challenging for less experienced surgeons

Various studies have shown the advantages of 3D navigation in complex spine surgery cases. Kalanchiam et al. showed the benefits of navigation guidance in managing C1–C2 pathologies. They used a 360-degree navigation guided approach to decompress the cervical spinal cord ventrally and also stabilize the upper cervical spine posteriorly (13). The authors highlighted the reduced surgical morbidity to the patients using this strategy. Similarly, Harel et al, in their study compared fluoroscopy guidance and navigation guidance for C1–C2 fixation in their series of 14 patients and concluded that navigation improves the safety of screw placement (14). Rajasekaran et al. studied the application of navigation guidance in children with complex cervical spine deformities and reported that using navigation, pedicle screws could be inserted accurately even in deformed vertebrae. They observed that out of 51 cervical pedicle screws, no screws had a critical pedicle breech, and only six screws (11.7%) had a non-critical breech. They concluded that pedicle screws could be efficiently placed irrespective of the pedicle width morphometrics (15). Similarly, Verma et al. in their meta-analysis included 23 studies with 5,992 pedicle screws and revealed a significant benefit in terms of accuracy of computer navigation-assisted screw placement compared with freehand screws (16). Scheufler et al. in their study reported an accuracy rate of 99.3% with the use of navigation in cervical pedicle screw insertion (17). Thus, with the use of an intra-operative navigation system and better 3D visualization, instrumentation in complex, intricate, and abnormal spine scenarios like in our case would be performed with more efficiency, thereby improving patient outcomes.

However, 3D based navigation platforms are not without limitations. The overall radiation exposure to the patient is high from these CT navigation systems, especially when repeated often during the surgical procedure (18). This causes concerns of radiation safety for the patient during the surgery. The possibility of motion artefacts does also exist, as the error rates can be high due to movement of the patient during the procedure. The accuracy of the tracking system also varies based on the distance of the tracker from the surgical site. All these should be carefully considered during the surgical procedure. One another important limitations of widespread application of 3D-CT navigation is the cost of installation (2). Smaller hospitals and Spine Centers with lesser volume of patients cannot afford to adapt this technology in day-to-day practice. However, in the long run, studies have shown to minimize the overall cost of the surgery by minimizing complication rates and improved patient safety (19, 20).

## Conclusion

The integration of 3D-CT navigation in complex spine surgeries like occipito-cervical and upper cervical pathologies not only enhances the precision and accuracy but also improves the overall safety of the procedure minimizing complications. The overall radiation exposure is also reduced considerably, thereby enabling the surgical team to perform the surgery with less occupational hazard. Considering the potential advantages of these navigation systems and further improved robotic technology, they would be a significant addition to improving the standard of care in spine pathologies. The technology effectively bridges the experience gap and enables the early career surgeons to perform complex surgeries with ease and confidence.

## Data Availability

The original contributions presented in the study are included in the article/Supplementary Material, further inquiries can be directed to the corresponding author.
